# Cationic covalent organic framework nanosheets as the coating layer of commercial separator for high-efficiency lithium-sulfur batteries

**DOI:** 10.1016/j.heliyon.2024.e36083

**Published:** 2024-08-10

**Authors:** Delong Ma, Xiaonan Tang, Aimin Niu, Xiupeng Wang, Mingchun Wang, Rongzhou Wang

**Affiliations:** School of Chemistry and Chemical Engineering, Shandong University of Technology, Zibo, 255000, PR China

**Keywords:** Covalent organic frameworks nanosheets (CONs), Battery separators, Batteries, Modified separator, Lithium-sulfur batteries

## Abstract

Ion-selective separators, are crucial and in high demand for maximizing the performance of lithium-sulfur (Li–S) batteries, which can conduct lithium ions while efficiently blocking polysulfides. However, commercial separators cannot effectively block the shuttle of polysulfides after multiple cycles due to their large porosity and easy dissolution, resulting in a reduced battery life. Herein, a covalent organic framework nanosheets (CON) ion-coated separator is prepared on the commercial separator. Due to the smaller pore size of CON-TFSI compared to polysulfides, the CON-TFSI modified separator can effectively block the polysulfide from migrating across the separator. By incorporating this innovative functional layer, Li–S batteries demonstrate outstanding performance. In a Li–S battery featuring a sulfur loading of 0.6 mg/cm^2^, it attains an initial discharge specific capacity of up to 891.9 mA h g^−1^, and exhibits the capacity retention of 54.6 % after 500 cycles at a current density of 0.2 C. This work offers a fresh perspective on the advancement of high-performance membranes for Li–S batteries.

## 1 Introduction

Lithium-sulfur (Li–S) batteries are widely acknowledged as highly promising contenders for next-generation energy storage systems. This recognition is primarily attributed to the cost-effectiveness of sulfur, its environmentally friendly nature, the high theoretical capacity (1675 mA h g^−1^), and a substantial specific energy density (2600 Wh kg^−1^) [[1], [2], [3], [4], [5], [6], [7], [8]]. Within Li–S batteries, separators play a crucial role by preventing short circuits between the anode and cathode, preventing the shuttle effect of polysulfides and promoting the migration of lithium ions [9]. Consequently, the characteristics of separators emerge as a critical determinant of Li–S battery performance. However, challenges arise during the cycling of Li–S batteries, including the dissolution of polysulfide intermediates and the significant consumption of electrolytes [[10], [11], [12]]. These issues contribute to several problems such as low sulfur utilization, rapid capacity fade, and a shortened cycle life.

To mitigate the dissolution of polysulfides, employing battery separators made of porous polymers has proven to be an effective strategy [[13], [14], [15], [16], [17], [18], [19], [20]]. However, conventional battery separators, typically composed of porous polymers like the commercially available polypropylene (PP) or polyethylene separators, fall short in inhibiting the diffusion of polysulfides, thus resulting in the internal short circuits within the battery. Hence, customizing the physical and chemical properties of the separator has proven to be an effective strategy for enhancing the performance of Li–S batteries. The implementation of an additional barrier layer, accomplished by coating conventional separators, has been suggested as a straightforward and direct method to mitigate the shuttle effect in Li–S batteries [[21], [22], [23]]. Recent advancements have shown the promising developments in the utilization of various functional materials, including nanostructured carbon, metal oxides, sulfides, and MXenes [24,25]. These materials are incorporated into separators to trap sulfur species through physical and/or chemical adsorption mechanisms. Nevertheless, these modified separators have exhibited drawbacks, such as inducing significant interfacial resistance and impeding the transport of metal cations. Therefore, an ideal separator should not only effectively facilitate the transportation of metal cations but also efficiently prevent the shuttle effect of polysulfides.

Covalent organic frameworks (COFs) constitute a rapidly growing class of crystalline porous polymers formed by organic building blocks linked via covalent bonds [[26], [27], [28]]. These versatile materials find applications in diverse fields such as gas storage and separation [[29], [30], [31]], drug delivery [[32], [33], [34]], sensor [35,36], catalysis [37,38], and energy storage [[39], [40], [41], [42]]. Moreover, the multifunctional properties of COFs can be effectively tailored by modifying the monomer structure or through ion exchange. Importantly, the inherently ordered channels of COFs play a crucial role in facilitating the transport of Li-ions and mitigating the undesirable phenomenon of polysulfide shuttling. Within the existing literature, COFs-based separators have demonstrated their efficacy as suitable coating for enhancing the cycle life of Li–S batteries [[43], [44], [45], [46], [47], [48]].

Herein, the cationic covalent organic framework nanosheets (CONs) were synthesized by incorporating cationic moieties into the structure of COF material [49,50]. The cationic CONs were subsequently employed as a coating material for modifying the separator of the Li–S battery. The inclusion of cations within the COF serves to effectively dissociate the ion pair of the Li salt, resulting in a higher concentration of freely mobile Li^+^ ions and, thus, enhancing Li^+^ conductivity. When chloride (Cl^−^) anions were substituted with bis(trifluoromethane) sulfonimide anions (TFSI^−^) in the ordered channels, the CON-TFSI was successfully synthesized. This modification enhanced Li^+^ transport properties compared to Cl^−^ anions. With these advantageous attributes, the CON-TFSI-modified separator significantly improved the performance of the Li–S battery with a sulfur loading of 0.6 mg/cm^2^. Following this modification, an impressive initial discharge-specific capacity of up to 891.9 mA h g^−1^ was achieved, with 487.4 mA h g^−1^ remaining even after 500 cycles.

## 2 Experimental

### 2.1 Synthesis of CON–Cl

CONs were synthesized according to a previously reported procedure. Specifically, 0.2 mmol of Tp (42 mg) and 0.2 mmol of TGCl (28 mg) were combined in a 10 mL Pyrex tube. A combination of dioxane and water in a ratio of 2:0.6 mL was introduced into the tube, followed by sonication of the resulting mixture for 20 min. Subsequently, the mixtures in the Pyrex tube underwent degassing under liquid N_2_ (77K) through freeze-pump-thaw cycles for three repetitions using a Schlenk line (with N_2_). The Pyrex tubes were then hermetically sealed under a static vacuum using an alcohol torch. Sealing the tubes under vacuum conditions resulted in optimal yields and crystallinity for CONs. The reaction mixture, enclosed in a sealed Pyrex tube, was kept at 120 °C for a duration of 3 days. Subsequently, brown-colored CONs precipitated from the solution. The obtained product was extensively washed successively with DMAc, water, and acetone, then dried overnight at 90 °C, yielding approximately 56 % of the isolated product.

### 2.2 Synthesis of CON-TFSI

The CON–Cl powder was dispersed and stirred in a 20 wt% LiTFSI aqueous solution (100 mL) at 30 °C to facilitate ion exchange. The solution was refreshed every 48 h for a total of three cycles. After completion, the solid product was obtained through centrifugation and subjected to multiple washes with deionized water and ethanol. This resulting material was then referred to as CON-TFSI.

## 3 Results and discussion

**Fig. 1:**
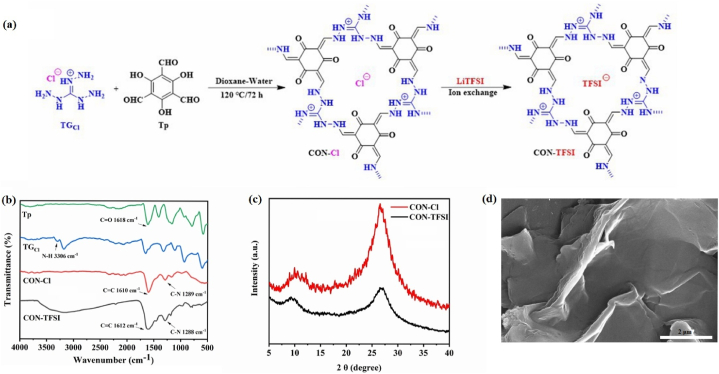
(a) Synthetic scheme of the CON-TFSI; (b) FTIR spectra of Tp, TGCl, CON–Cl, and CON-TFSI; (c) FTIR spectra of CON–Cl and CON-TFSI; (d) SEM image of CON-TFSI.

CON-TFSI and the intermediate CON–Cl were prepared by conducting a Schiff-base condensation reaction employing conventional methods, as shown in Fig. 1a. Specifically, 1,3,5-triformylphloroglucinol (Tp) and triaminoguanidinium chloride (TG_Cl_), as both monomers, underwent sonication in a sealed Pyrex tube. The mixture was then heated in a dioxane/water mixture (2:0.6, by volume (mL)), resulting in the formation of CON–Cl. Subsequently, CON-TFSI was obtained through an anion exchange process between CON–Cl and lithium bis(trifluoromethane)sulfonimide (LiTFSI).

The Fourier transform infrared (FTIR) spectrum of Tp revealed a prominent absorption band corresponding to the C=O stretching mode at 1618 cm^−1^. This particular absorption feature was conspicuously absent in the FTIR spectra of CON–Cl and CON-TFSI. Instead, the FTIR spectra of CON–Cl and CON-TFSI exhibited stretching bands at 1610-1612 cm^−1^ (C=C) and 1289-1288 cm^−1^ (C–N), indicative of the formation of a keto-enamine linkage (Fig. 1b). The crystal structures of both CONs were elucidated using simulated powder X-ray diffraction (PXRD) patterns (Fig. 1c). In both CONs, prominent peaks at 9.7° were clearly identified in the PXRD patterns, indicating low crystallinity and originating from the (100) plane. Simultaneously, a wide peak at 2θ = ∼28° appeared in the PXRD patterns of both CON–Cl and CON-TFSI, indicating the presence of weak π-π stacking between the vertically aligned 2D layers. The observed low crystallinity in the CONs can be attributed to the interaction between the loosely bound anions and the intrinsic positive charge of the Tp monomer. This interaction disrupts the π-π stacking interactions between the structural layers, contributing to the overall reduced crystallinity in the materials. The scanning electron microscopy (SEM) image revealed that CON-TFSI displayed a nanosheet morphology characterized by loose and irregular features with a smooth surface texture (Fig. 1d).

**Fig. 2:**
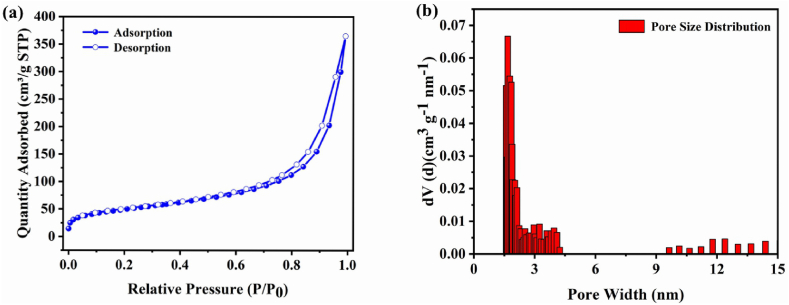
(a) N_2_ adsorption and desorption isotherms of CON-TFSI; (b) the pore size distribution of CON-TFSI.

The Brunauer-Emmett-Teller (BET) surface area of CON-TFSI was ascertained to be 174 m^2^ g^−1^, mainly attributed to factors like insufficient layer stacking, a small pore diameter, and blockage of pores by the counteranions (Fig. 2a). The pore size is smaller than that of polysulfides (Li_2_*Sn*, 4 ≤ n ≤ 8) but larger than that of lithium ions. As a result, lithium ions can pass through the CON-TFSI coating layer, while soluble polysulfides are blocked in the cathode region. Pore size distributions were computed using nonlocal density functional theory (NLDFT). Consistent with prior findings, distinct and well-defined pore size distributions were not observed due to the absence of a structured channeled pore arrangement and the blocking of pores by subsequent counteranions (Fig. 2b).

In addition, the thermogravimetric analysis (TGA) curves indicated that both CON-TFSI and CON–Cl remained stable and did not undergo decomposition before reaching 200 °C in N_2_ (Fig. S1). Additionally, SEM images of CON–Cl revealed the presence of distinct sheets (Fig. S2). The TEM images of CON-TFSI and the mappings of the elements (C, N, O, F, and S) in CON-TFSI reveals the morphology and elemental distribution of CON-TFSI (Fig. S3). The BET surface area of PP membrane was ascertained to be 33 m^2^ g^−1^ (Fig. S4), significantly less than that of CON-TFSI. The contact angle of the CON-TFSI coated separator (7^o^) is lower compared with the pristine PP (52^o^) (Fig. S5). X-ray photoelectron spectroscopy (XPS) full-spectrum scans revealed that the chlorine (Cl) signal almost disappeared following ion exchange in CON-TFSI (Fig. S6). Utilizing a commercial Celgard substrate, the functional layer for CON-TFSI was engineered through a method employing vacuum-assisted self-assembly. Notably, the CON-TFSI coating layer remained intact even after undergoing a folding test, indicating its robust mechanical strength (Fig. S7). The SEM images of the cross-section of the modified membrane also indicate the successful modification of the PP membrane by CON-TFSI (Fig. S8).

**Fig. 3:**
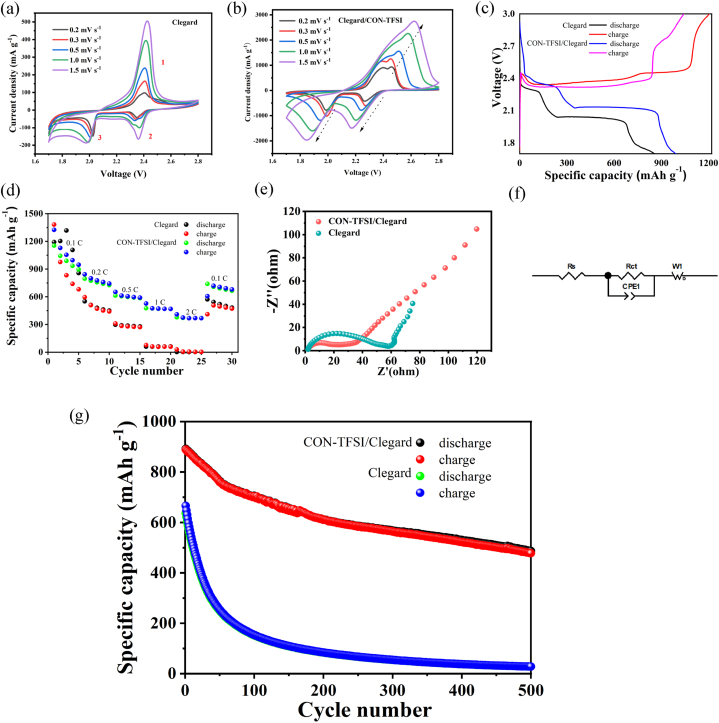
CV curves with different sepators at different scanning rates ranging from 0.2 to 1.5 mV s^−1^: (a) unmodified PP; (b) coated with CON-TFSI; (c) The galvanostatic charge-discharge curves of different separators at a current density of 0.1 C; (d) The rate performance of two kinds of separators at a current densities of 0.1 C, 0.2 C, 0.5 C, 1.0 C and 2.0 C; e) The EIS spectra of different sepators before cycling; (f) The corresponding equivalent circuit diagram; (g) The cycle life of different separators at a current density of 0.2 C.

Afterward, we further investigated the electrochemical performance of the CON-TFSI coated separator in a Li–S battery and the results were shown in Fig. 3. The batteries were labeled as CON-TFSI/PP battery and PP battery, respectively. Fig. 3a-b shows the cyclic voltammetry (CV) curves of Li–S batteries with two types of separators, scanned at a rate of 0.2 mV s^−1^, within a voltage range of 1.7–2.8 V. The CV results display two distinct reduction peaks and one oxidation peak typical of Li–S batteries, indicative of the sulfur species transformation. The cathodic peak around 2.3 V denoted the reduction reaction converting insoluble sulfur into soluble polysulfides (Li_2_*Sn*, n = 4–8). Simultaneously, the cathodic peak around 2.0 V signaled the transformation of soluble Li_2_S_x_ into insoluble Li_2_S_2_/Li_2_S. The anodic peak around 2.4 V corresponds to the reverse process of the reduction reaction, where insoluble Li_2_S_2_ and Li_2_S theoretically oxidize to form long-chain polysulfides and further oxidize to elemental sulfur [51]. Compared with the uncoated sample, the modified sample shows multiple oxidation peak coupling phenomena, indicating that after improving the ability to transport lithium ions, multiple interfacial side reactions may occur simultaneously at the positive electrode. Additionally, from Fig. 3a-b, it can be seen that the CON-TFSI/PP battery exhibits higher peak currents and larger curve areas for oxidation-reduction in both samples, indicating a high oxidation-reduction rate.

The observed experimental phenomenon aligns precisely with the galvanostatic charge-discharge profiles of both the CON-TFSI/PP battery and the PP battery during their initial cycle. This agreement is evident in Fig. 3c, where the tests were conducted at a current density of 0.1C within the voltage range of 1.8–2.8 V. Both batterys showed two-stages during the discharge process and one-stage during the charge process, which were also demonstrated on the cyclic CV curves. Two potential plateaus of CON-TFSI/PP battery were observed around 2.3 V and 2.1 V in the discharge profile. This stage denoted the conversion of elemental sulfur (S_8_) into extended-chain polysulfides Li_2_S_x_ (4 ≤ x ≤ 8), and the subsequent reduction of these extended-chain polysulfides into S_2_^2−^/S^2−^. Moreover, the rate performances of the Li–S batteries using the CON-TFSI modified PP separator and PP separator were estimated at various current densities (0.1 C, 0.2 C, 0.5 C, 1 C, and 2 C) (Fig. 3d). Obviously, the experimental results indicate that the PP battery showed a much lower capacity, and poor rate performance. As expected, this finding aligns with the outcomes obtained from electrochemical impedance spectroscopy (EIS), a crucial metric for assessing the electrochemical kinetics and conductivity of devices. According to the EIS plots (Fig. 3e) and corresponding equivalent circuit diagram (Fig. 3f), the impedance spectrum is characterized by a distinctive structure comprising a semicircular segment in the high-frequency region and an oblique straight line in the low-frequency region. Typically, the intersection point of the semicircle signifies the solution resistance (R_s_) of the electrolyte, while the semicircle itself reflects the charge.

Transfer impedance (R_ct_). Meanwhile, the linear segment is associated with the Warburg impedance (W_o_), indicating ion diffusion throughout the charge and discharge processes. The relevant parameters are obtained according to the equivalent circuit in Fig. 3f batterys employing the CON-TFSI modified separator exhibit a Rct of 16.48Ω, a value notably lower than that observed in batterys employing the PP separator, which recorded an Rct of 47.63Ω, due to the high conductivity of the CON-TFSI modified layer and the high capacity retention of the electrolyte, which accelerates the transfer of electrons and ions to the positive sulfur electrode.

To further investigate the long-term cycling stability, the CON-TFSI/PP battery was tested at 0.2 C for 500 cycles (Fig. 3g). For the cycling performance, the PP battery gave the lower initial capacities (637 mAh g^−1^), of which 4.3 % (27.9 mA h g^−1^) of the initial capacities were retained after 500 cycles. Nevertheless, the CON-TFSI/PP battery shows significantly improved electrochemical performance, and gave a high initial capacity of 891.9 mA h g^−1^, and a specific capacity of 487.4 mA h g^−1^ was also obtained after 500 cycles and a remarkable capacity retention was 54.6 %, showing a greatly exbatteryent cyclic performance in CON-TFSI/PP battery. Therefore, cycling performance indicated that the CON-TFSI/PP battery was obviously superior to the PP battery. Based on the analysis of the above electrochemical performance results, the modified material was coated on the PP separtor of Li–S battery to effectively inhibit the shuttle of polysulfide and improve the transport efficiency of lithium ions.

## 4 Conclusion

In summary, we utilized a cationic CON layer to modify the separator in Li–S batteries, thereby enhancing the performance of Li–S batteries. The CON-TFSI structure adsorbed TFSI^−^ anions, reducing pore diameter and creating a sieving mechanism for polysulfides. As a result, Li–S batteries with CON-TFSI/PP separators demonstrated enhanced rate performance and specific capacity, and stable cycling stability. This study advances Li–S battery performance by addressing the polysulfide shuttle issue and improving Li + transport, highlighting the potential of customizable framework materials in innovative energy storage systems.

## Data and code availability

Data will be made available on request.

## CRediT authorship contribution statement

**Delong Ma:** Writing – review & editing, Writing – original draft, Project administration. **Xiaonan Tang:** Project administration. **Aimin Niu:** Writing – review & editing, Formal analysis. **Xiupeng Wang:** Methodology, Investigation. **Mingchun Wang:** Writing – review & editing, Writing – original draft, Project administration. **Rongzhou Wang:** Writing – original draft, Project administration.

## Declaration of competing interest

The authors declare that they have no known competing financial interests or personal relationships that could have appeared to influence the work reported in this paper.
