# Supplementary material for: Cationic covalent organic framework nanosheets as the coating layer of commercial separator for high-efficiency lithium-sulfur batteries
Source: Heliyon. 2024 Aug 10;10(16):e36083. doi: 10.1016/j.heliyon.2024.e36083 (PMC11369462; doi:10.1016/j.heliyon.2024.e36083)
Supplement: Multimedia component 1 [file mmc1.pdf]

Supplementary Information

Cationic Covalent Organic Framework Nanosheets as the Coating Layer of Commercial Separator for High-Efficiency Lithium–Sulfur Batteries

Delong Ma [1], Xiaonan Tang [1], Aimin Niu, Xiupeng Wang, Mingchun Wang *, Rongzhou Wang *

School of Chemistry and Chemical Engineering, Shandong University of Technology, Zibo 255000, P.R. China

[1] These authors contributed equally to this work.

* Corresponding authors. E-mails: 1751018153@qq.com (M. Wang), rongzhouwang@sdut.edu.cn (R. Wang)

1. Preparation of Sulfur Cathode

In this paper, S cathode material was prepared by a typical melting diffusion method. First, grind S and Super P in a ratio of 60:40 for 20 min until evenly mixed. Next,the mixture is transferred to a tubular furnace where it is heated for 12 hours under the protection of argon gas at 155 °C. Preparation of the positive electrode sheet: The mixed material, Super P and PVDF are dissolved in NMP at a ratio of 8:1:1 and ground evenly. The paste is then coated on aluminum foil and dried in a vacuum oven at 60 °C for 12 hours. The surface density of the positive polar plate is about 0.6 mg/cm^2.

2. Method of preparation of CON-TFSI/PP

CON-TFSI and PVDF were milled homogeneously in the ratio of 9:1. Next, the well-mixed slurry was coated onto a polypropylene separator (PP, Celgard 2400). The thickness of the coated slurry was 50 um. Subsequently, it was placed in a vacuum drying oven and dried at 60°C for 12 hours. After drying, the prepared CON-TFSI/PP were cut into small 16 mm discs for use using a slicer.

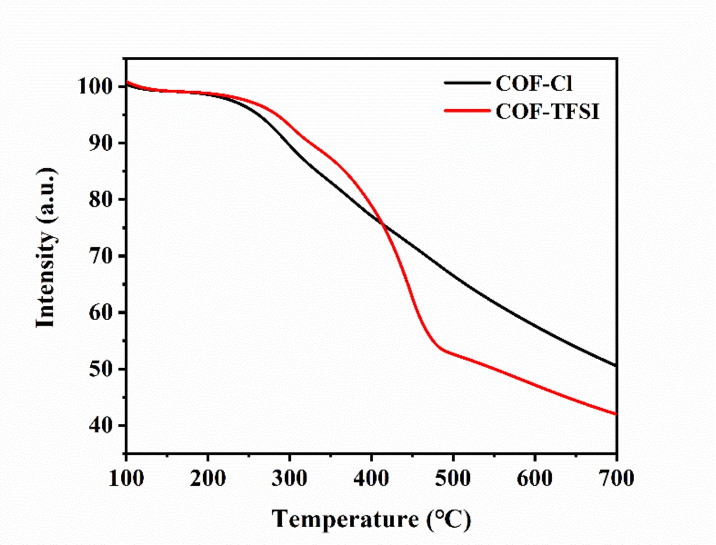

Fig. S1 TGA curve of COF-Cl and COF-TFSI.

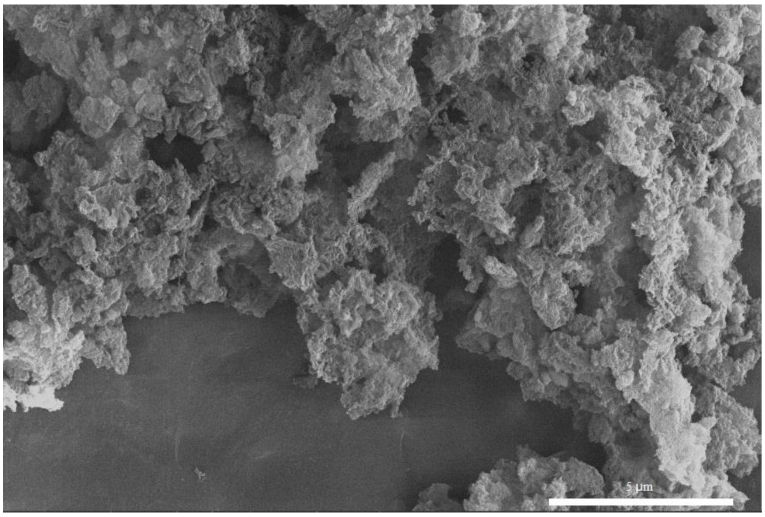

Fig. S2 SEM image of COF-Cl.

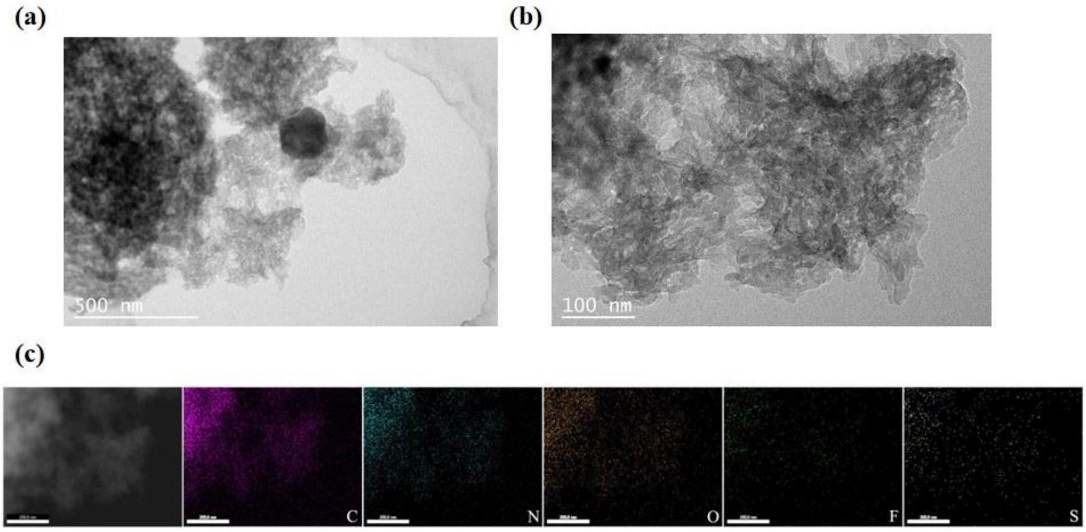

Fig. S3 (a) and (b) TEM images of CON-TFSI; (c) Mapping of the elements (C, N, O, F, and S) in CON-TFSI.

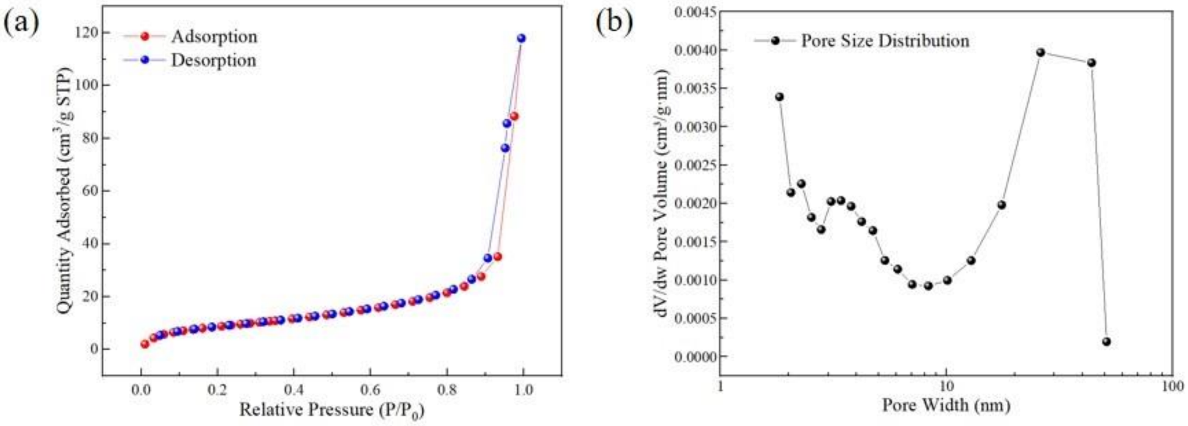

Fig. S4 (a) N_2 adsorption and desorption isotherms of PP membrane (b) the pore size distribution of PP membrane.

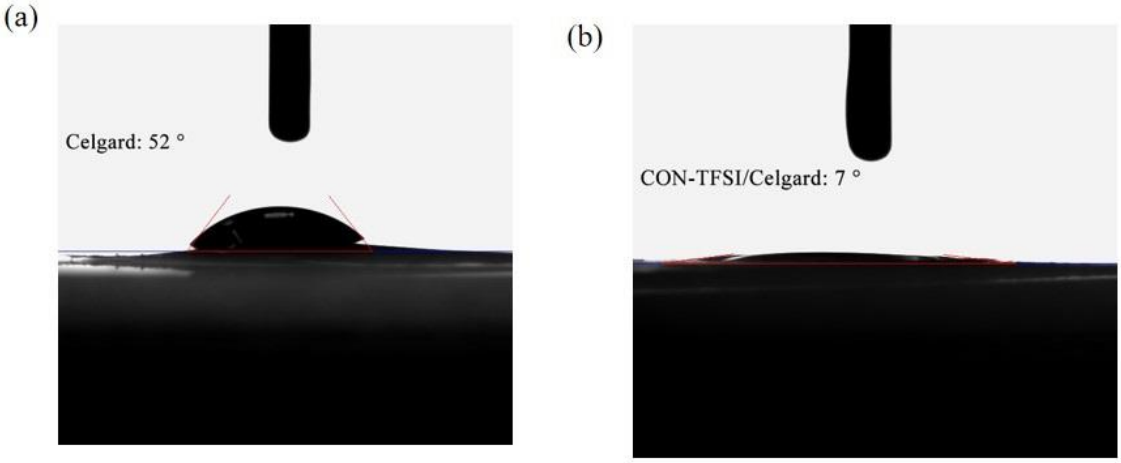

Fig. S5 (a) Initial contact angle of PP separator; (f) Initial contact angle of CON-TFSI coated separator.

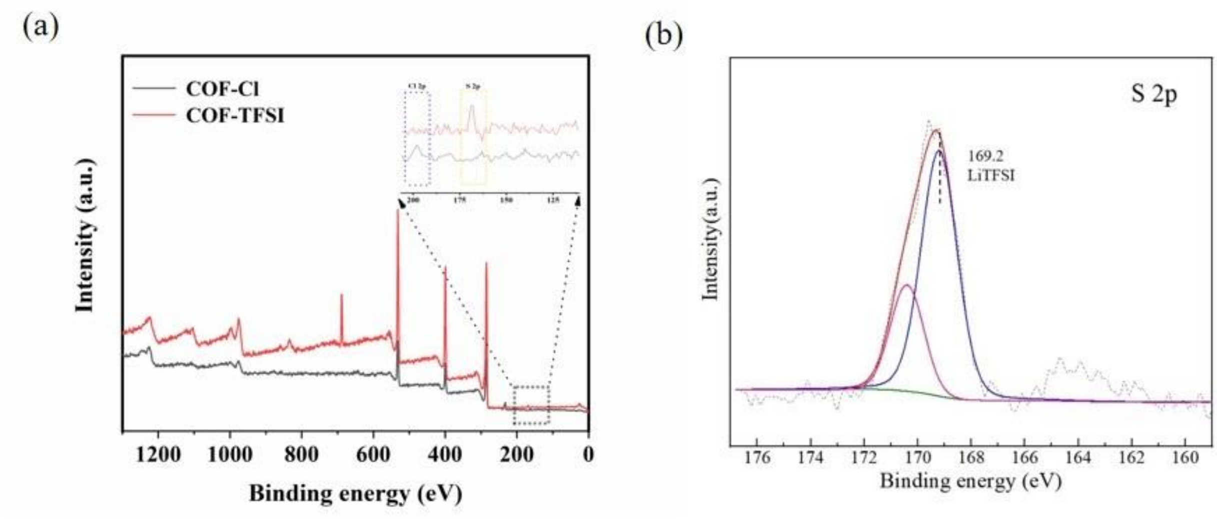

Fig. S6 (a) X-ray photoelectron spectra of COF-Cl and COF-TFSI; (b) S 2p spectra.

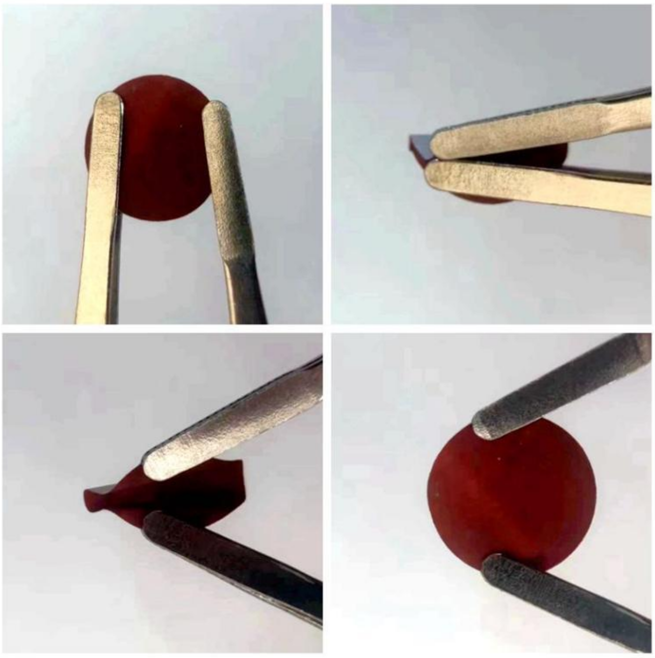

Fig. S7 Photographs of the COF-TFSI/Celgard.

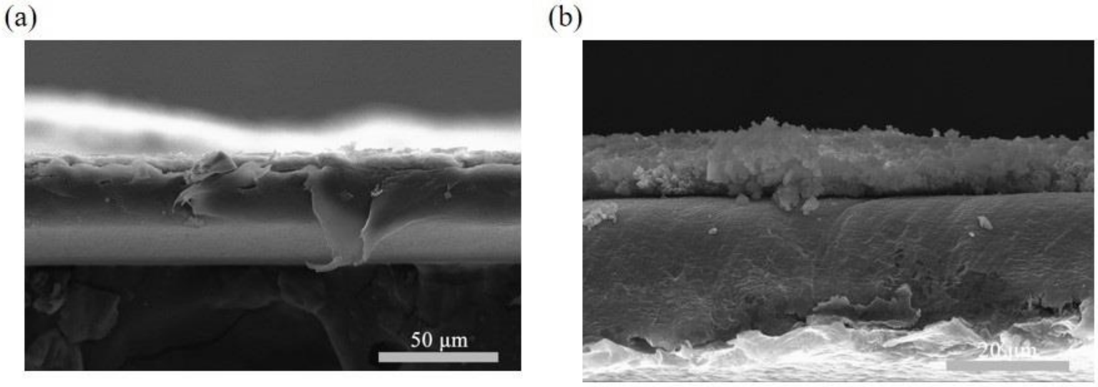

Fig. S8 (a) and (b) The SEM image of the CON-TFSI-modified PP membrane.
